# Disclosure of Children's Positive Serostatus to Family and Nonfamily Members: Informal Caregivers in Togo, West Africa

**DOI:** 10.1155/2011/595301

**Published:** 2011-07-07

**Authors:** Ami R. Moore, David Williamson

**Affiliations:** Department of Sociology, University of North Texas, 1155 Union Circle no. 311157, Denton, TX 76203, USA

## Abstract

This study examined the structural constraints to disclosure of children's positive serostatus among informal caregivers to family and nonfamily members in Togo. It drew on two data sources, one qualitative and the other quantitative. Qualitative data showed that caregivers cautiously disclosed child's positive serostatus for fear of being stigmatized and discriminated against as well as to protect the children from being stigmatized. Binary regression analyses revealed that different factors influenced reasons for disclosure of a child's serostatus. For instance, while caregivers' serostatus and number of children significantly influenced disclosure for financial support, disclosure of a child's serostatus for spiritual support was strongly affected by education and religion. These results shed light on factors and reasons for disclosure among caregivers. This knowledge is important because different types of programs and advice should be given to caregivers with specific reason(s) for disclosure instead of creating a “one-size-fits all” program for all caregivers.

## 1. Introduction

Disclosure of positive HIV serostatus beyond the caregiver-patient dyad has been shown to vary according to many factors including education [1, 2], source of medical care [3], condom and alcohol use [4], religion [5], number of sexual partners [4], ethnicity [6], socioeconomic status [7], relationship to the one to whom the disclosure is made [6–10], and gender [4]. Although findings are somewhat inconsistent, most have found that disclosure is most likely when the target (one to whom the disclosure is made) is intimate or a close family member, when the discloser is a woman, is of lower socioeconomic status, is acculturated rather than a recent immigrant [6], uses condoms but does not use alcohol heavily before sex, and has fewer sexual partners. 

Of the factors inhibiting disclosure, the fear of stigmatization is among the most common [4, 6, 11]. Also commonly noted are the fear of abandonment, the loss of economic support, and the fear of violence from an intimate partner [3, 9]. 

One means of explaining the anomalies and inconsistencies in findings has been proposed by Brandt et al. [12], who argue that contextually grounded research aimed at illuminating individual-level strategic decisions is the best way to understand disclosure patterns. This methodological warning is consistent with the concerns of Vanlandingham et al. [13] that population-based studies of AIDS overlook the contextually rich community and individual-level realities in which disclosure decisions are made.

In this research, we attempt to take the debate on disclosure of positive serostatus further by looking at both reasons for disclosure and factors affecting reasons for disclosure. Thus, we examined the structural constraints to disclosure by investigating the individual and contextualized realities that constrain disclosure by analyzing qualitative data as well as quantitative survey data on factors that affect reasons for disclosure from the same population in Togo, West Africa. We propose that as more such triangulated research is conducted and cross-group variations are analyzed, a more satisfying model of disclosure may be derived [14, 15]. Using a qualitative approach, we first asked caregivers of children living with HIV and AIDS a list of all the people to whom they disclosed child's serostatus and the reasons for disclosure. We believe that because of stigma and discrimination, people disclose positive serostatus for different reasons and knowing such reasons and factors that affect disclosure may help HIV service providers and stakeholders in devising sound programs that will be helpful to both informal caregivers of people living with HIV and AIDS and people with HIV and AIDS. Conceptual themes and phrases created from the qualitative study were used to create a questionnaire used to survey 210 caregivers.

The hypotheses tested in the study are

1. factors affecting the reasons why caregivers disclose child's serostatus will vary,
2. there will be a gender effect on disclosure of child's serostatus,
3. education (Education here is a proxy for socioeconomic status) will have a negative effect on disclosure of child's serostatus.

## 2. Setting

Togo is a low income country located in West Africa. With an estimated population of 6.6 million in 2008, Togo has an agricultural economy and most Togolese are engaged in subsistence agriculture. The estimated per capita income was $770 in 2006 [16]. Togo has experienced a decline in its HIV and AIDS prevalence rates. From a high as 5% in the early 2000s, HIV and AIDS prevalence rate was estimated at 3.3% among adults aged 15 to 49 in 2006 [17]. The estimated pediatric infections for children aged 0 to 14 years was 10,000 in 2007 [18]. However, AIDS was the leading cause of death among Togolese of all ages [17]. Although there is a significant unmet need between people who need antiretroviral therapy (ART) and those who actually receive it, more and more Togolese living with HIV and AIDS have been on ART [19]. Consequently, they are living longer and healthier lives. Although HIV and AIDS campaigns are relatively prevalent in Togo, HIV and AIDS related stigma and discrimination against people living with HIV and AIDS are common. Thus, people affected by and/or infected with HIV and AIDS do not generally disclose this to others [10, 20].

## 3. Methods

This study drew on two data sources, one qualitative and the other quantitative. Because of the sensitive nature of the study, we asked the directors at three centers that provide services to people with HIV and AIDS and their families to recruit primary caregivers of children aged 0 to 15 who were HIV positive or had AIDS. After explaining the study protocol to the directors, they selected five of their employees who had at least a Bachelor's degree in social sciences to help with the study. We used a convenience sampling method to select participants. To be included, a participant had to be a current primary caregiver and also had used the centers' services in the past. Those who met the eligibility criteria and were willing to participate in the study completed informed consent forms. Participants were compensated $10. The five employees were trained by the first author for two days. As part of their training, they also watched the author conduct the first 10 qualitative interviews and later the first 10 surveys. To ensure consistency in data collection, the five employees met with the first author once a week and she checked their interviews and questionnaires. Data were collected from July to December, 2006 in Lomé and the surrounding towns.

### 3.1. Qualitative Data Collection

First, through in-depth interviews, the experiences of 30 caregivers were assessed. Caregivers were asked to give a detailed description of all the things they did from dawn to dusk to and for the seropositive child. They were also asked to describe ways they managed caregiving demands, for example, what they did specifically when the child had ups and downs. They were asked to list all the people to whom they disclosed the serostatus of the child and the reason(s) for disclosure. They were also asked to list all the family members and others that provided any kind of support and specify what the person did. This study focused solely on factors that influence reasons for disclosure. Interviews were in French or Ewe (one of the native languages in Togo). They were recorded and lasted on average about 60 minutes. Interviews were transcribed verbatim. Then, recurrent themes and phrases were classified and grouped into categories.

### 3.2. Quantitative Data Collection

Second, from the conceptual themes and phrases generated from disclosure questions, we created questions that reflected disclosure-related issues which were consequently administered to 210 caregivers. Whenever possible, we tried to retain verbatim phrases for the questions. The questions went through a series of pretests and revisions. A set of Likert-style frequency descriptors was used for all response options (4—completely agree; 3—agree; 2—somewhat agree; 1—disagree). For this study, we dichotomized the responses into (1) at least somewhat agree and (2) disagree. Participants who disagreed with any of the statements in the questionnaire did not disclose the serostatus of the child to anyone while those who at least somewhat agreed had disclosed the child's serostatus to at least one person. The disclosure questions were (a) did you disclose serostatus of child to people who can financially help you? (b) did you disclose serostatus of child to people who can emotionally help you? (c) did you disclose serostatus of child to people who can spiritually help you? (d) did you disclose serostatus of child to your own family members? (e) did you disclose serostatus of child to nonfamily members? (f) did you disclose serostatus of child to your own children?

## 4. Results

### 4.1. Qualitative Data

Two main concepts explained disclosure of child's serostatus among caregivers: fear of being stigmatized and discriminated against as a caregiver of a child with HIV and concern of stigma directed toward the child. All of the caregivers had disclosed child's serostatus to people who could provide some support such as medical professionals or HIV service providers. However, they cautiously disclosed to others who were neither healthcare professional nor HIV service providers. For instance, Kofi (names are pseudonyms), who was caring for his three-year-old nephew, stated when asked about people to whom he had disclosed the child's serostatus:

Only one of my three sisters, my wife, and my mother know about it (child's serostatus). I did not tell anyone else because I'm afraid if people know about it, we will be asked to move out of the house…. I have to say that health professionals who care for the child also know about it.

When asked how he knew for certain that the reaction of people would be negative, he added:

Even my wife does not like him (seropositive nephew) very much. She does not like to give him his medicines. I care for him myself to make sure that he eats well and takes his medicines.

Another HIV-positive mother, Ayaba, who had lost her husband earlier made the comments below when asked about the people who knew the serostatus of her 14-year-old daughter:

I went to my family when she was really sick, but no one helped me. Thus, as they did not help, I did not want to disclose our serostatus to them…. This is a disease of shame and we will disgust them. The only people that know about our serostatus are the HIV service providers. Our landlord and his children help us sometimes but they don't know what is ailing us. I'm afraid that if they know they will sack us from the house…. I have also disclosed to 2 ministers.

Later, when asked about why she disclosed their serostatus to the ministers, Ayaba stated that she needed spiritual support and counsel. 

Another respondent, a seropositive mother of a six-year-old child, stated the following when asked about people to whom she had disclosed the child's serostatus and the reasons for disclosure:

My parents passed away and I have only one sister but she does not know about it. When she asked about my child's illness, I tell her my child has internal wounds…. My other children also tell people that their brother has malaria or internal wounds. The children don't know about their brother's serostatus either…. They are children and at times they argue and fight. I don't want them to know as they may throw it (positive serostatus) in his face.

Louise, a 46-year-old widow who was caring for her 9-year-old granddaughter made the comments below when asked about people to whom she had disclosed the serostatus of the child:

Only my mother, my sister, and the child's mother know about this (child's positive serostatus). My sister advised me not to tell anybody else because people are afraid of the illness (AIDS). My mother and sister help me care for her…. I told her teacher at school that my grand daughter has asthma. Even when other people ask question about my grand daughter's illness, I tell them the same thing.

Furthermore, although HIV-related campaigns are more prevalent in Togo relative to the past few years and people are being educated about the means of HIV transmission, the stigma and discrimination are still present. Thus, some of the people affected/infected know that having HIV or AIDS does not necessary lead to death as it used to in the past, but they still do not disclose positive serostatus. Afiwa, who was caring for her 13-year-old niece, stated the following when asked about people to whom she had disclosed the child's serostatus:

In my own family, only my mother knows about it…. AIDS is like leprosy in the 1700s. One day, there will be a cure for it. Personally, I have seen people with AIDS who are healthy and also all the HIV programs that are on TV tell me that AIDS is simply an illness. It is a matter of being informed and knowing about the illness (AIDS). However, people with HIV are still stigmatized and discriminated against.

About 65% of the caregivers felt the same way as Afiwa. They were very hopeful that ARVs would make the children or both of them stronger and healthier, but because of HIV-related stigma and discrimination, they concealed the positive serostatus from people that they did not trust to keep disclosure a secret or would not contribute anything positive to help their situation.

### 4.2. Quantitative Data

Although 210 caregivers were surveyed, the sample was reduced to 201 because of missing cases. Table 1 presents the descriptive characteristics of caregivers and odds ratios of caregivers' disclosure of child serostatus. Caregivers were mainly females (73%) with a mean age of about 42 years. The mean number of years of education was about 9 years. The majority of the caregivers were married (57%). Caregivers had on average 3 children. Forty-four percent of the caregivers reported being seronegative, 33% were seropositive, and the remaining 23% did not know their serostatus. More were Catholic than any other religious denomination (about 47%). 

The odds of disclosure of a child's serostatus for financial support were not significantly influenced by caregivers' age, sex, marital status, religion, or education. However, caregivers' serostatus and number of children showed significant relationships with this type of disclosure. Reporting an HIV-negative serostatus reduced caregivers' disclosure for fear of losing financial support by 58% [(1 − 0.416) × 100] compared to caregivers who were seropositive. Number of children reduced the likelihood of disclosure for financial support. In fact, the odds of disclosure were reduced by 22% by the presence of each additional child. 

Caregivers' serostatus and marital status were predictors of disclosure of child serostatus for emotional support. As in the case of disclosure for financial support, the odds of disclosure were 65% [(1 − 0.347) × 100] reduced for caregivers who reported a seronegative status relative to caregivers who were HIV positive. Additionally, caregivers who were married were about 2.1 times more likely to disclose for emotional support compared to those who were not married. 

Disclosure of a child's serostatus for spiritual support was significantly affected by education and religion. Compared to caregivers with a higher education, having a primary education increased the odds of disclosure for spiritual help by 19.4 times. Also, Protestant caregivers were 18.2 times to disclose for spiritual help relative to their counterparts with a traditional religion.

Disclosure to one's own family was influenced by age and education. Relative to caregivers who were 50 and older, the odds of disclosure to family members were lowered by 85% [(1 − 0.148)∗100] and 76% [(1 − 0.239)∗100], respectively, for caregivers who were less than 30 and those who were 30 to 49 years old. Furthermore, disclosure was about five times higher for caregivers with a primary education and 5.6 times higher for those with a secondary education compared to those with a higher level of education. 

Only one variable significantly influenced disclosure of a child's serostatus to nonfamily members. Compared to caregivers who were HIV positive, reporting a negative serostatus reduced the odds of disclosure to nonfamily members by 62% [(1 − 0.377)∗100]. 

Disclosure to caregiver's own children was predicted by age, sex, serostatus, marital status, and number of children. Relative to older caregivers, the odds of disclosure to one's own children were reduced by 80% [(1 − 0.198) × 100] for caregivers who were 30 to 49 years old. Additionally, compared to women, being a male reduced the odds of disclosure by 83.5% [(1 − 0.165)∗100]. Caregivers who reported a negative serostatus were about 5.6 times more likely to disclose child's serostatus to their own children relative to those who were HIV positive. Married caregivers were 3.8 times more likely to disclose serostatus of the child to their own children. Having an additional child increased the odds of disclosure by about 1.7 times.

## 5. Conclusion

This study examined the structural constraints to disclosure of children's positive serostatus among informal caregivers to family and nonfamily members in Togo. Qualitative data from informal caregivers of children with HIV and AIDS revealed that caregivers cautiously disclosed positive serostatus of the children for whom they were giving care. Additionally, the caregivers experienced HIV-related stigma and discrimination by association. They were also worried about stigma and discrimination directed toward the child. Consequently, they took proactive measures by disclosing only to people who they trust and/or could provide them with support. 

In fact, the question of disclosure becomes more complicated when the decision maker about the disclosure is not the patient but his/her caregiver. Borrowing from Goffman [21, 22], caregivers may be viewed as part of the “performance team” that manages the image of the family unit. The adult caregiver has to manage information about the serostatus of the patient as well as the image of the family and of the caregiver him or herself. Also, because of misunderstanding regarding HIV modes of transmission, people may stigmatize and discriminate against both caregivers and children who are HIV positive. Studies have shown that misunderstanding about HIV modes of transmission is prevalent in Africa. Castle [23] found in Cote d'Ivoire a common belief that AIDS is transmitted by young girls having sexual encounters with the dogs of white people and in Mali many believe that one can become infected by urinating in a place where someone who is seropositive has already urinated or by sharing their clothes.

Furthermore, as hypothesized, factors that affect the reasons why caregivers disclose child's serostatus vary. Also, there is a gender effect on disclosure of child's serostatus. However, this effect was significant only for disclosure to caregivers' own children. Education also had a significant influence on disclosure for spiritual support and to one's own family members. 

Additionally, several comments can be made about the findings of the quantitative data. While caregivers' serostatus significantly affected the odds of disclosure of child's positive serostatus for financial support, emotional support, and disclosure to one's own children and nonfamily members, it did not affect the odds of disclosure for spiritual support, nor disclosure to one's own family members. One may infer that caregivers who reported an HIV negative status were not as pressed for emotional and financial support as their counterparts who were HIV positive. Thus, they were less likely to disclose in order to obtain these kinds of support. However, these caregivers were more likely to disclose a child's positive serostatus to their own children compared to caregivers who were HIV positive. This may be explained by the fact that children tend to ask questions about their HIV-infected status or that of their siblings and/or relatives [24, 25]. Therefore, caregivers who reported seronegative status may be more willing to disclose a child's serostatus and answer questions about their own serostatus relative to caregivers who are seropositive. Also, having an additional child increased the odds of disclosure to one's own children. Perhaps, the more children caregivers had, the more these children asked questions about the health of the child with HIV/AIDS. Caregivers may then end up disclosing child's serostatus.

Married caregivers were more likely to disclose a child's serostatus for emotional support and to their own children possibly because they had the support of their spouse who may encourage them to disclose. Furthermore, while education did not significantly affect disclosure of a child's serostatus for financial support, emotional support, disclosure to nonfamily members and to one's own children, caregivers with a primary education were likely to disclose for spiritual support and those with a primary and a secondary levels of education disclosed more to their own family members relative to their counterparts with a higher education. Perhaps, because of the low socioeconomic status of caregivers with lower levels of education, they might not have the necessary skills to deal with caring for a child with HIV/AIDS. Consequently, these caregivers were more likely to disclose the child's serostatus for spiritual support, as well as disclosing to one's own family in order to garner resources needed to care for and meet the needs of an HIV-positive child. It is possible that their low socioeconomic status may have precluded any fear of stigmatization that might come from disclosure. 

Age significantly affect disclosure to own family members and to own children. Relative to their counterparts who were at least 50 years old, caregivers who were less than 50 years old were less likely to disclose possibly because older people may strongly feel stigma and/or the impact of HIV-related stigma as in the case of some older American PLWHA who were more stigmatized because of their age [26]. With regard to disclosure for spiritual support, Protestant caregivers were more likely to disclose. Protestant religious groups, especially the conservative ones, tend to have a more community-oriented environment [27] and also emphasize faith (charismatic) healing more than non-Protestant religions [28]. Thus, Protestant caregivers were more likely to disclose child's serostatus.

There are several limitations about this study. First, the sample is small and nonrandomized. Second, the sample came from only 3 HIV centers among over 30 centers in Togo. Thus, the findings may be interpreted with caution as there may be biases in our sample population. Third, because of the small sample size, some of the confidence intervals are quite large. 

Despite the limitations of the study, some implications of stigma and discrimination on disclosure have been uncovered. Significant social realities affect disclosure of a child's serostatus among informal caregivers. In fact, because of stigma and discrimination, caregivers cautiously disclose a child's seroctatus. Disclosure is also influenced by different reasons and factors. As found in this study, factors that affect one's odds of disclosure vary by reasons why one may want to disclose in the first place. Thus, it is important that researchers examine reasons for disclosure in order to ascertain factors that may affect disclosure among people infected/affected by HIV, as these factors may vary by demographic and socioeconomic characteristics. Furthermore, knowing the reason(s) why caregivers want to disclose should help service providers at HIV/AIDS centers and HIV/AIDS program planners devise appropriate programs for caregivers. This knowledge is important because different types of programs and advice should be given to caregivers with specific reason(s) of disclosure instead of creating a “one-size-fits all” program for all caregivers.

## Figures and Tables

**Table 1a:** (a)

Explanatory variables			Disclosed for financial support			Disclosed for emotional support		
				95% CI			95% CI	
Descriptive statistics			Odds ratio	Lower	Upper	Odds ratio	Lower	Upper
Age group	*N*	Mean = 41.66						
Less than 30	26	(13%)	0.358	0.072	1.791	0.487	0.102	2.326
30 to 49	124	(62%)	0.390	0.145	1.048	0.496	0.190	1.295
50 and above	51	(25%)	1^1^			1		

Sex								
Male	55	(27%)	2.042	0.387	4.986	1.421	0.596	3.389
Female	146	(73%)	1			1		

Serostatus of caregiver								
Do not know	47	(23%)	0.697	0.2226	2.190	0.692	0.230	2.083
HIV negative	88	(44%)	0.416	0.180	0.963	0.347	0.152	0.792
HIV positive	66	(33%)	1			1		

Marital status								
Married	114	(57%)	1.342	0.640	2.813	2.135	1.026	4.440
Not married	87	(43%)	1					

Educational attainment		Mean = 8.62						
No formal education	30	(15%)	2.448	0.514	11.655	0.847	0.176	4.065
Primary education	69	(34%)	3.643	0.987	13.450	2.473	0.668	9.147
Secondary education	76	(38%)	2.217	0.644	7.631	2.368	0.679	8.255
Higher	26	(13%)	1			1		

Religion								
Catholic	95	(47%)	0.992	0.240	4.094	0.944	0.225	3.963
Protestant	86	(43%)	1.651	0.396	6.882	0.928	0.221	3.899
Islam	9	(5%)	0.277	0.032	2.414	0.744	0.097	5.704
Traditional/other	11	(5%)	1			1		

Number of children	201	Mean = 3.08	0.778	0.638	0.949	0.894	0.740	1.081

**Table 1b:** (b)

Explanatory Variables	Disclosed for spiritual support			Disclosed to my own family members		
		95% CI			95% CI
	Odds ratio	Lower	Upper	Odds ratio	Lower	Upper

Age group						
Less than 30	4.718	0.839	26.537	0.148	0.028	7.795
30 to 49	2.373	0.778	7.233	0.239	0.081	0.706
50 and above	1^2^			1		

Sex						
Male	0.803	0.372	2.071	1.207	0.495	2.947
Female	1			1		

Serostatus of caregiver						
Do not know	1.292	0.372	4.482	0.480	0.149	1.543
HIV negative	0.700	0.287	1.711	0.613	0.261	1.441
HIV positive	1			1		

Marital status						
Married	1.430	0.639	3.202	0.921	0.428	1.980
Not married	1			1		

Educational attainment						
No formal education	2.717	0.231	31.929	3.814	0.808	18.014
Primary education	19.442	2.051	184.335	5.060	1.364	18.773
Secondary education	7.566	0.835	68.587	5.586	1.611	19.369
Higher	1			1		

Religion						
Catholic	4.712	0.485	45.745	1.364	0.328	5.676
Protestant	18.192	1.868	177.199	1.139	0.275	4.725
Islam	1.447	0.064	32.798	1.179	0.153	9.075
Traditional/other	1			1		

Number of children	1.047	0.837	1.309	0.853	0.702	1.037

**Table 1c:** (c)

Explanatory Variables	Disclosed to nonfamily members			Disclosed to own children		
		95% CI			95% CI	
	Odds ratio	Lower	Upper	Odds ratio	Lower	Upper

Age group						
Less than 30	0.652	0.124	3.429	0.010	0.028	7.795
30 to 49	0.722	0.253	2.067	0.198	0.056	0.691
50 and above	1^3^			1		

Sex						
Male	1.433	0.577	3.556	0.165	0.052	0.529
Female	1			1		

Serostatus of caregiver						
Do not know	0.718	0.223	0.223	3.011	0.680	13.338
HIV negative	0.377	0.154	0.924	5.577	1.821	17.077
HIV positive	1			1		

Marital status						
Married	1.370	0.630	2.978	3.832	1.341	10.951
Not married	1			1		

Educational attainment						
No formal education	1.833	0.259	12.951	1.335	0.157	11.314
Primary education	3.229	0.587	17.762	1.068	0.194	5.865
Secondary education	3.137	0.597	16.478	1.464	0.287	7.479
Higher	1			1		

Religion						
Catholic	1.615	0.289	9.032	0.509	0.087	2.978
Protestant	2.178	0.395	12.025	0.474	0.080	2.828
Islam	0.501	0.033	7.602	0.184	0.012	2.811
Traditional/other	1			1		

Number of children	0.955	0.783	1.166	1.685	1.269	2.293
